# The Care of the Teeth

**Published:** 1897-06

**Authors:** F. H. Funston


ARTICLE X.

### THE CARE OF THE TEETH.

BY F. H. FUNSTON, M. D.

No meal should ever be partaken of without immediately thereafter rinsing and washing the mouth with clean, clear water, if nothing else, and obviously the addition of some pleasant antiseptic, like euthymol, is preferable. So, too, this act should be succeeded by a thorough scrubbing of the teeth with a moderately stiff brush, passed both laterally and perpendicularly over front and back surfaces alike; not even the most microscopic quantity of food should be allowed to remain in the interstices of the teeth or about the gums. Such a measure, if carefully carried out with the aid of a strictly antiseptic dentifrice, will prove not only a means of warding off offensive breath and the many maladies common to the gums and teeth, but will speedily eradicate tartar and retracted gums.

During the last half-century dentifrices have multiplied by thousands, each presenting its own peculiar claim. Some are really valuable; others are harmless; not a few are dangerous. In the latter connection, too great stress cannot be laid upon the fact that any article intended to be utilized for the toilet of the teeth, and which presents an acid or markedly alkaline reaction, is to be regarded with suspicion; it may be added that a number in the market are neither more or less than dilute solutions of spirit of salt (muriatic acid), which is almost rapid in its action upon enamel, and moreover promotes decay and tends to produce offensive exhalations. Others are little more than pleasantly flavored soap; but if the latter ingredient is good and pure it cannot be considered objectionable. Tooth powders, too, which sometimes accompany fluid dentifrices, must also be looked upon with suspicion, as they not infrequently contain ingredients that may prove detrimental. And it may here be remarked that those applications for the teeth which are warranted to immediately whiten are always dangerous, inasmuch as they rely upon strong chemicals for their action.

A recent improvement in this line is euthymol tooth paste, which, as its name indicates, depends in large measure for its value upon euthymol, a preparation that has long been employed by surgeons wherever perfect antisepsis was desired, and has moreover deservedly gained universal popularity because of its freedom from danger except to germ life.

To the mind of the writer this preparation warrants specific mention inasmuch as it offers the ideal of a dentifrice in that it is at the same time a powerful antiseptic, reasonably detergent, modest in price, pleasant in odor, and exceptionally grateful to mouth and gums, while last, but not least, its use affords a positive protection against foul breath and other conditions peculiar to the mouth that lead to retraction and softening of the gums, staining of enamel, formation of tartar; it is likewise a reasonably certain guarantee against a number of diseases which gain entrance to the human organism through germs in the mouth and digestive organs.—*Popular Science News.*

